# Exploring Local Aspects for a Culturally and Contextually Adapted Family Therapy Training in Cambodia

**DOI:** 10.1111/famp.70014

**Published:** 2025-02-16

**Authors:** Bernhild Pfautsch, Matthias Ochs, Bouyheak Lim, Celia Jaes Falicov

**Affiliations:** ^1^ Department of Social Work Fulda University of Applied Sciences Fulda Germany; ^2^ Psychology Practice Sombok Psychology Phnom Penh Cambodia; ^3^ University of California, San Diego San Diego California USA

**Keywords:** Cambodia, cultural adaptations, family therapy education

## Abstract

The goal of this study was to further local knowledge production and the ground‐up cultural and contextual competencies needed for family therapy training and practice in Cambodia. First, expert interviews were conducted with Cambodian and international mental health professionals with several years of professional experience in Cambodia (*N* = 25; 72% Cambodian, 52% female). Current characteristics of Cambodian family life and related problems were identified, as well as aspects of the current reality of mental health services in this Southeast Asian nation, which continue to be counted among the low‐ and middle‐income countries (LAMICs). Second, in a group discussion with four Cambodian trainer experts in family therapy, the cultural and contextual competencies to be developed in the training of Cambodian family therapists were identified. The competencies for incorporating religious beliefs, respecting hierarchical family structures/expressing respect, and competencies for dealing with the cultural imperative of silence/face‐saving appear to be significant. For the trainees, it is precisely here that areas of tension were described—triggered by collusion of their own value systems with those implicit in Western concepts. These moments of alienation should be carefully registered for reflection. The experience of dissonance in relation to certain content and procedures points precisely to the need for adaptation by local experts for the development of culturally appropriate curricula.

## 1 Introduction

The internationalization of professional knowledge from the field of family therapy calls for reflection to detect a potentially colonizing agenda (Arnett 2008; Polanco 2016). For the development of culturally sensitive family therapy curricula, knowledge as well as formal learning contexts must be viewed as socially constructed and relative, influenced by multiple systems of privilege and oppression (McDowell and Shelton 2002). In addition, the multifaceted perspectives of historical, sociocultural, and political determinants of mental health must be included. Thus, local traditions of thought and narratives about suffering, distress, and lived experiences, as well as their preferred healing practices, are crucial references for the development of locally appropriate family therapy and corresponding training curricula. Therefore, family theories should be placed in their Western context of origin. In the sense of a decolonizing approach, its implicit values, traditions, and intentions should be considered in their foreignness in the current local context (Polanco 2016).

The Cambodian context was explored in the present study using the Multidimensional Ecological Comparative Approach (MECA) (Falicov 1995, 2014a). Local cultural value preferences in Cambodia relevant to family therapy as well as the impact of sociopolitical dimensions and contextual stressors for local families could be described and then contrasted with Western concepts. Similarly, the competency framework developed by the Center for Outcomes Research and Effectiveness (CORE), explicitly the list of basic competencies for systemic therapy (Pilling et al. 2010; Stratton et al. 2011), was used for this study. As a competency list of Western systemic therapy, CORE served as a frame of reference for comparison to explore systemic therapeutic competencies relevant in Cambodia and to determine cultural fit and the resulting need for attunement.

Through the theoretical reference of MECA (Falicov 1995, 2014a), which in its logic of cultural comparison clearly opposes an unreflective Western determinism toward the object of research, an approach of decolonial research practice (cf. Bermúdez et al. 2016) was realized. The intention of the present study is to support local, ground‐up knowledge production outside of the Western paradigm.

Currently, systemic family therapy approaches are gaining interest in Cambodia and have already been introduced and practiced primarily by nongovernmental organizations (NGOs) in outreach settings in Cambodia (Miller et al. 2019; Seponski et al. 2014). Yet until now, comprehensive academic training has been lacking in this area (Seponski and Jordan 2018). The first Postgraduate Certificate in Family Therapy and Systemic Practice was launched by the Royal University of Phnom Penh in 2017. The program was established for professionals working in various settings of individual and family counseling in Cambodia (GIZ‐Cambodia 2017; RUPP 2018). To date, three cohorts have completed this training. Four Cambodian psychologists (MA) underwent a train‐the‐trainer program and will be responsible as experts for the implementation and further development of the course in the future.

### 1.1 Background of the Study

Cambodia experienced a massive collective trauma during the Khmer Rouge regime from 1975 to 1979, with an estimated 1.5–3 million people falling victim to this genocide (Seponski et al. 2019). The executions and torture were often carried out by young people, many of whom are now themselves parents and grandparents (Miles and Thomas 2007). Many mental health professionals were killed by the Khmer Rouge regime, which resulted in almost no resources available to meet the trauma of genocide and civil war (Stewart et al. 2010). The development of the necessary infrastructure and human resources for basic mental health care remains a major challenge to this day due to the collapse of the education and health systems during the civil war (Jegannathan et al. 2015). High prevalence rates of anxiety disorders (24.7%), depression (16.7%), and post‐traumatic stress disorder (7.6%) determine the needs in mental health (Seponski et al. 2019).

#### 1.1.1 Family Therapy Training in Cambodia

The first education in counseling based on Western concepts took place in 1995 with the training of Cambodian professionals in the field of mental health. Counseling and family therapy were included among the taught approaches at that time (Somasundaram et al. 1999). However, a more comprehensive academic training in family therapy has been lacking to date (Seponski and Jordan 2018). The *Postgraduate Certificate in Family Therapy and Systemic Practice* offered by the Psychology Department of the Royal University of Phnom Penh (RUPP) since 2017 is so far the only opportunity to obtain a professional specialization in family therapy (GIZ‐Cambodia 2017).

#### 1.1.2 Competencies of Cambodian Therapists

As a frame of reference for professional maturity in family therapy training, core competencies have been the focus of Western educational institutions for around 30 years (Nelson et al. 2007). Competencies include the ability to act in a self‐organized and creative manner, as these qualities are in demand when it comes to complex action requirements. Motivation to act is generated from internalized values that are associated with emotions (Erpenbeck et al. 2017). Thus, competence development is steered by values (Erpenbeck and Rosenstiel 2007). Reflection and self‐assurance about their own values and those of their clients, therefore, play an important role for trainees (cf. Jordan et al. 2019). Training that is consistently linked to these values can promote the development of culturally appropriate family therapy skills.

#### 1.1.3 Adaptation to Content and Service Delivery

Collectivist ideals have received little attention in dominant academic research traditions of individualistic culture (Bermúdez et al. 2016). Therefore, making Western theories relevant to Cambodian therapy practice and avoiding colonization and cultural conflict require awareness of inherently different values and perspectives (Seponski et al. 2020) and an emphasis on the legitimacy of local knowledge, behaviors, and concerns of families (Bermúdez et al. 2016).

In a recent study looking at the culturally sensitive implementation of Western therapeutic models in Cambodia, Seponski et al. (2020) conclude that in order to meet the demands of family therapy training in the cultural milieu of Cambodia, it is important to either adapt therapeutic interventions to the cultural context or to create those ground up. The purpose of this study is to take up these concerns.

### 1.2 Purpose of the Study

The aim of this study was to gather information from mental health professionals working with Cambodian families regarding their views about relevant competencies that are needed for their work and thus also have relevance for the design of corresponding training curricula. For this purpose, the participants were interviewed as experts in order to gain their knowledge of therapeutic practice with families, their current conditions, and the problems they face. The aim was to discover appropriate socioculturally attuned therapeutic approaches. The study took place in Phnom Penh, and the Cambodian experts interviewed referred to their experiences with families in urban and rural areas in a differentiated way. The available concepts of Western family therapy had to be critically examined in terms of their cultural fit (McGoldrick and Giordano 2005; Miller and Fang 2012; Tseng et al. 2019). This included locally favored or realistic treatment settings and modalities of access to counseling and family therapy services as documented by previous studies conducted in Cambodia (Alfredsson et al. 2017; Miller et al. 2018; Seponski et al. 2020).

## 2 Method

The aims of the study led to the following research questions: (a) How do key concepts of family therapy (Falicov 2014a) appear in the cultural context in Cambodia and which problems are named by the interviewed professionals as relevant for family therapy? (b) Under what conditions is family therapy currently feasible in Cambodia? (c) Which core competencies in family therapy are prioritized by therapists in Cambodia? (d) What implications does this have for family therapy training in the country?

The last research question relates to the results of the previous questions. For this reason, the present study follows a sequential triangulation design (Flick 2004). After a criteria‐based case selection, expert interviews were conducted first, followed by a group discussion. Qualitative content analysis with inductive categorization (Mayring and Fenzl 2019) was applied for data analysis.

### 2.1 Participants

A criterion‐guided case selection (Kelle and Kluge 2010) was used to select 25 (13 female) mental health professionals with work experience in Cambodia with both urban and rural clients by the following selection criteria: (a) professional background/academic training in the psychosocial field, (b) several years of work experience in Cambodia, and (c) currently working in this field in Cambodia. As mentioned in the Introduction section, there is currently no university training in family therapy, which is why the group of participants was expanded to include mental health professionals. However, of the 18 Cambodian professionals interviewed, 13 had completed the RUPP's postgraduate certificate in family therapy.

Because there were two subgroups among the interviewees, Cambodian professionals and international professionals, a central variable (cultural origin) was varied in order to increase the explanatory power of the study (Gläser and Laudel 2010). The sociodemographic data of the participants are presented in Table 1.

**TABLE 1:** Sociodemographic characteristics of participants.

Characteristic	Cambodian experts (*n*)	International experts (*n*)
Gender		
Female	9^a^	4
Male	9	3
Years of professional experience in Cambodia in the field of Mental Health	5–29	10–22
Professional background	Social work, psychology, psychiatry	Counseling, psychology, art therapy, child psychiatry

*Note:* To our knowledge, there were neither Cambodian nor international family therapists with many years of experience working in Cambodia at the time of the interviews.

^a^ Including the trainers from the group discussion.

The following considerations were decisive for limiting the number of cases: In order to answer the research questions, it was important to have as many different perspectives as possible; at the same time, for practical research reasons, the effort for the survey and evaluation had to be feasible (Gläser and Laudel 2010). For expert interviews, a manageable number of cases of 20–30 interviews is recommended (Meuser and Nagel 2002). This recommendation corresponds to the number of 26 interviews conducted, 25 of which could be evaluated.

The four Cambodian trainers for family therapy took part in the group discussion. They all have a master's degree in psychology and completed the RUPP's postgraduate certificate in family therapy and then a train‐the‐trainer program in 2019. To our knowledge, this group contained the entire population of trained family therapy trainers in the country. The expertise of these four trainers from their perspective as educators was fundamental, particularly in answering the fourth research question. They were able to contribute their local perspective on the content and teaching formats of the training. The form of collaborative group discussion was chosen to explore their collective body of knowledge related to the training (Przyborski and Riegler 2018).

### 2.2 Procedure

The Multidimensional Ecological Comparative Approach (MECA) is a systemically oriented postmodern framework for culturally adapted family therapy in theory, research, clinical practice, and training (Falicov 1995, 2014a, 2014b). The domains of MECA are migration, ecological context, family organization, and family life cycle. Because this study focuses on local families in Cambodia, migration aspects were not in the foreground of interest; therefore, only the last three of the above domains have been utilized to explore the relevant fields of reference for family therapy in Cambodia.

Core competencies, in the sense of outcome‐based education, provide the frame of reference for family therapy education in Western contexts (Nelson and Graves 2011). The CORE competency framework (Pilling et al. 2010; Stratton et al. 2011) compiles competencies for family therapy work in European contexts. CORE served as a theoretical frame of reference for a comparative approach to develop competencies for the Cambodian needs elicited by MECA. CORE has been used similarly in Taiwan to identify shared competencies and those that are unique to the local context (Chao and Lou 2018).

**FIGURE 1:**
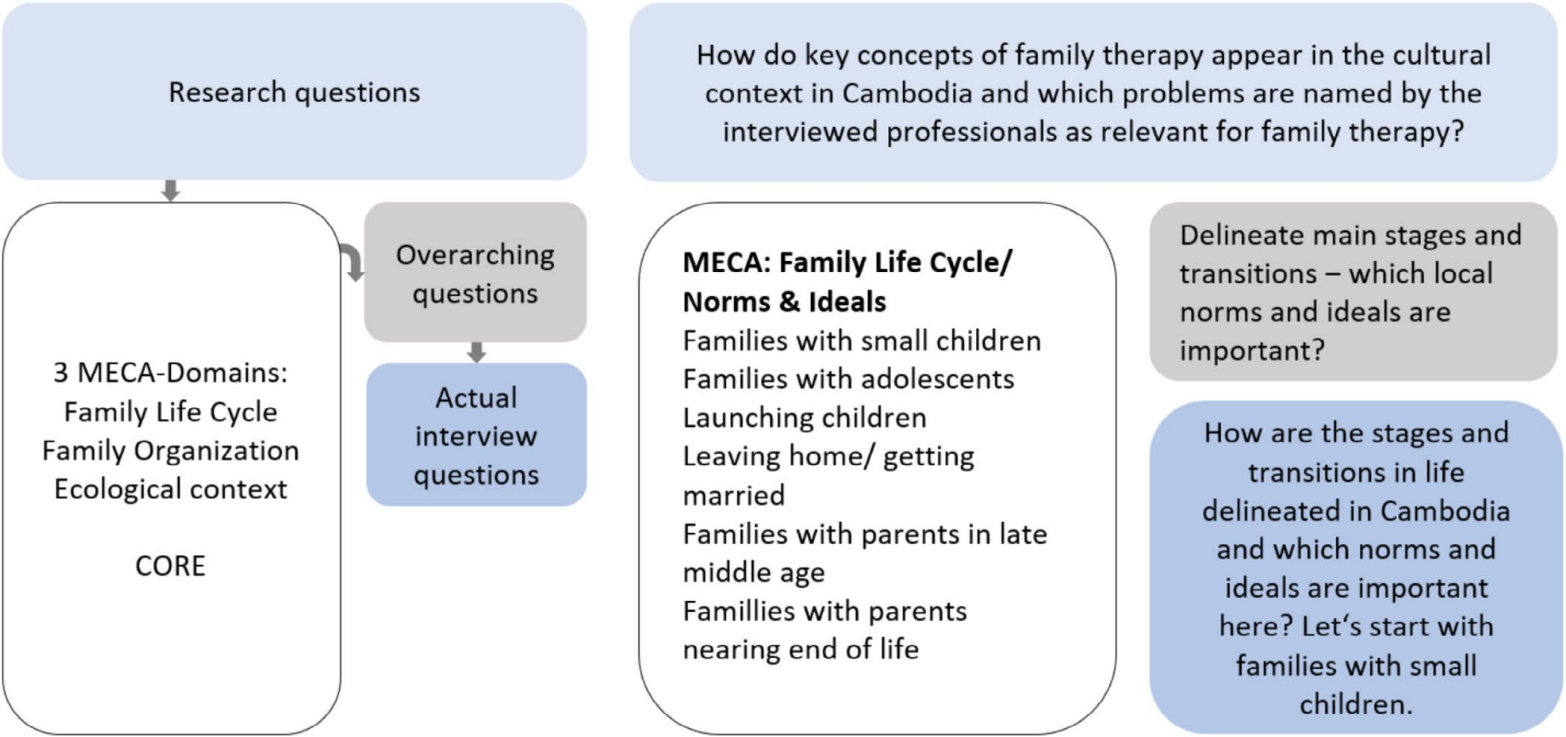
Procedure for the use of MECA and CORE in the development of the interview guide.

This study asks about the various aspects of Cambodian family life, the contextual conditions for family therapy, and the resulting therapeutic skills required locally. To answer these questions, an interview guide for semistructured interviews (Gläser and Laudel 2010) was developed by Bernhild Pfautsch (the guide is available upon request). The research questions were derived from the MECA model (Falicov 2014a). Key questions were then developed in relation to the aspects subsumed there, which in turn became the basis for formulating the actual interview questions. In the same way, the CORE basic competencies for systemic therapy (Pilling et al. 2010; Stratton et al. 2011) provided the theoretical reference for the last question in the interview guide (see Figure 1). The guide was cross‐checked for cultural sensitivity by a Cambodian counselor before conducting the interviews.

The CORE basic competencies were already known to the group discussion participants from their training. A summary of these competencies (initiate contact and undertake a systemic assessment; engage relevant members of the system; formulate goals; support networks and own capacities; work in a reflexive manner; use monitoring to promote change; manage endings) was presented to the group participants on a slide to stimulate discussion about these contents. The unique sociocultural aspects for a Cambodian family therapy found in the interviews are presented in Table 2. These were introduced to the group as further themes for discussion. The interactive group discussion aimed to stimulate the exchange of knowledge in relation to the training, which has been structured by shared experiences of the discussants (Przyborski and Riegler 2018). The selected participants in the group discussion have the specific perspective as local training experts; their perspectives expand the insights gained from the interviews. This explains the decision to supplement the interviews with this group discussion and thus to triangulate the methods in this study (Flick 2004).

**TABLE 2:** Significant local themes emerging from interviews.

Theme	Transcript segment
Saving Face as A Cultural Concept	28
Discrepancy between Idea of Equality and Hierarchical Family Organization	32
Expectations of Advice and Instructions from Therapists	50
Experience of Tension between (Western) Theory and Trainers' Cultural Values	72
Management of Uncertain Family Therapy Process Conditions	82

*Note:* The transcript comprised a total of 104 segments.

### 2.3 Data Collection

After receiving approval from the ethics committee of Fulda University of Applied Sciences, the interviews were conducted by Bernhild Pfautsch face‐to‐face in March 2019 at different locations in Phnom Penh and recorded as audio. All participants were known to the interviewer from previous work contexts, and trust had already been established. They were informed about the purpose of the research and gave their written consent. The choice of locations was based on the needs or suggestions of the interviewees. One interview with an international expert turned out to be unusable afterward due to strong noise interference. The interviews lasted an average of 77 min (range 35–106 min) and were conducted in English. Prior to the interview, the interviewees were informed about the research purpose, consent was obtained in writing, especially for recording, and the contact details of the interviewer were provided for possible later queries. The initial contact for the interviews was realized by e‐mail or directly in the meeting with former colleagues at the university.

A planned research visit of the first author to Cambodia in Spring 2020 could not take place due to the COVID‐19 pandemic. Therefore, the group discussion was conducted as a videoconference in July 2020. Due to the pandemic, online formats have become established in many social domains as well as in European research contexts. When using online formats, their specific form of communication must be reflected in comparison to the traditional communication of co‐presence (Przyborski and Wohlrab‐Sahr 2021). With group discussion conducted online, nonverbal communication may have diminished (Wirtz et al. 2021). As the group discussion progressed, however, it became fluid, lasting 63 min.

### 2.4 Data Preparation and Processing

The available audio recordings of the interviews and the video recordings of the group discussion were transcribed by the first author. Since the focus of the analysis was primarily on manifest content, a semantic content transcription system was chosen (Dresing and Pehl 2018). All names mentioned in the interviews and other references that could have led to identification were anonymized. For the analysis process, the data processing software MAXQDA (Kuckartz 2020) was used.

### 2.5 Data Analysis

**FIGURE 2:**
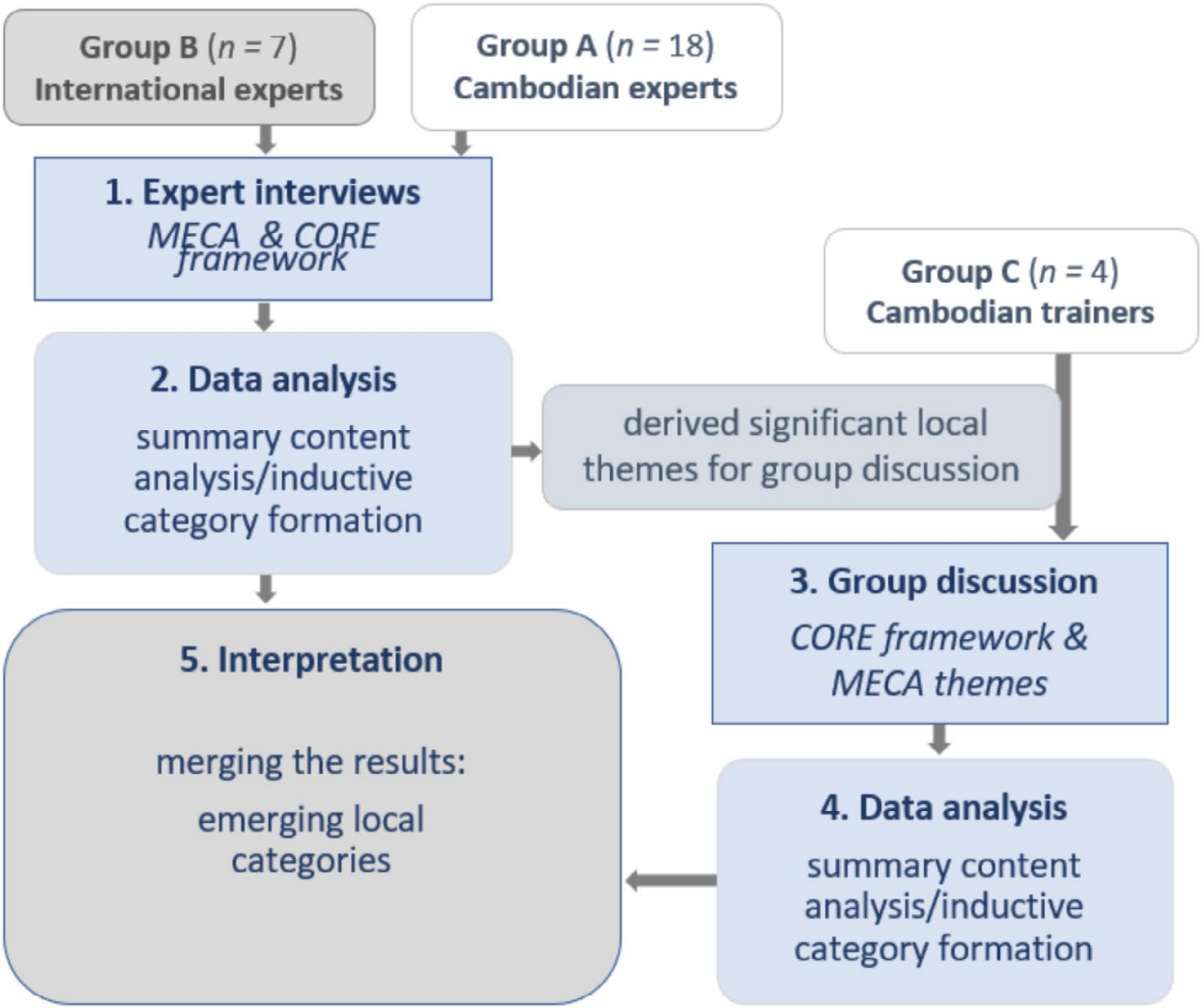
Research process flow.

Qualitative content analysis with inductive categorization (Mayring and Fenzl 2019) was applied to the interview data by the first author. First, main categories were deductively determined from the two theoretical references: MECA and CORE. In the following inductive step, subcategories were formed while working through the texts line by line to further differentiate the themes of the main categories. A revision of the categories took place after coding nine interviews, after hardly any new aspects appeared in the material (Mayring 2014). The category system now available was discussed by Bernhild Pfautsch with Mathias Ochs and among a collegial data analysis group at their university regarding its reliability and revised accordingly by Bernhild Pfautsch and finally used for all interviews. Significant derived local themes from this analysis were introduced as additional question stimuli in the group discussion (Figure 2).

The group discussion was analyzed in a similar way to the interviews. The results of the interviews and the group discussion were finally merged in the interpretation as newly emerging local categories (Figure 2). For the analysis of the group discussion, deductively derived main categories from the CORE Basic Systemic Competences (Pilling et al. 2010; Stratton et al. 2011), were applied to the text; these were then further differentiated by inductive category formation (cf. Mayring and Fenzl 2019).

As part of the sequential triangulation approach in this study, five themes (Table 2) that emerged from the analysis of the interviews and appeared relevant were introduced as question stimuli throughout the group discussion.

### 2.6 Intercoder Check

The intercoder check as a required step in this protocol of qualitative content analysis (Mayring and Fenzl 2019) was performed by Bouyheak Lim familiar with the culture. After the introduction of the category system, she fully coded one interview, which was then used to check intercoder agreement using the software's corresponding analysis tool. The results, primarily the nonmatches, were discussed in terms of intersubjective validation. Particularly in the case of inductive category formation, complete agreement could not be expected during the intercoder check; instead, the focus was on discussing the non‐agreements found (Mayring and Fenzl 2019). In this way, divergently assigned codes could be made comprehensible to the other side and fruitful for data interpretation.

## 3 Results

In accordance with the research questions mentioned above, in the following section, the (a) key concepts of family therapy that reflect current family life and the (b) contextual conditions for family therapy work in Cambodia are illustrated with brief excerpts from the findings. Knowledge of those societal conditions for services forms the basis for contextual competencies for the implementation of family therapy on a local level. The findings for (c) the resulting practice competencies are then presented in more detail. Finally, the significance of reflexive competencies as one of the (d) implications for family therapy training in Cambodia is exemplified. Pseudonyms were used for the persons quoted.

### 3.1 Family Life in Cambodia—Current Picture and Implicit Problems

Extended families live together in a hierarchical organization, which forms a large support network, in rural areas also as a village community. The international experts in our study emphasize this in particular and refer to the different family organization in Cambodia compared to the West, as Sally puts it:

There is a kind of norm of “you live with your parents or your grandparents or the uncles and the aunts—the whole system.” […] So, I think, compared to western culture where is much more a nuclear family unit I think, this extended family system here and also, the community system, especially when we go to the provinces were the community also takes care of—in the ideal sense—of children. I think, there is a much larger net, a support net for caretaking.

However, the findings of the study point to profound social and cultural change and provide evidence of trends in social transitions in which extended family values are being replaced by nuclear family values. Chenda expresses her concerns as follows: “The development of the country—and the development that goes SO FAST which doesn't give value to the well‐being, the quality time for the family and all that.”

#### 3.1.1 Conflicts due to Different Values Within the Family

In parenting, questioning established traditions that lead to conflicts. This creates several dilemmas for families: Contradictory claims, conflicts of loyalties, and parallel moral codes. Intrafamily tensions and conflicts were named when family members adopt new values or when within the family the old values are maintained in different proportions—especially in the field of parenting. Raksmey describes her position:

In some houses they discipline the children in one way but the older people the way they discipline is still a bit more traditional way. For example, like threatening in some way like that. It's also the conflict of the power as well because we are in the middle, for example, the couple in the middle, we have to listen to the older people.

The study identifies the resulting ambivalences for the younger generation to abandon traditional gender roles and embrace the new opportunities of education and economic success while shouldering financial responsibilities and meeting the cultural expectations of older family members. Panha expresses this with his statement: “Yeah, I think, that change is really frustrating for them (younger generation) because they have to fight with the traditional way. And some parents if they are understanding, they will accept it and some they will cut tie.” Other interviewees confirmed that family therapists should offer help in resolving such conflicts.

#### 3.1.2 Consequences of Economic Pressure

Young people leave the traditional structures—either with the privilege to study in the urban centers or less privileged as young labor migrants inside the country. Many grandparents are confronted with taking care of grandchildren on a large scale due to the current migration movements, as Raksmey expresses:

I feel sad, but I don't blame them, I put this as a responsibility of the society that they could not create enough jobs for them to be here. […] I was extremely sad when I talked to a few families who—especially one of them I remember the most—she had to take care of 11 grandchildren.

Intergenerational solidarity in extended families continue to retain great importance.

Further, in the data is described how the collective traumatization of an entire generation is observed to have a transgenerational impact and influence the parenting behaviors of parents and grandparents. Narith expresses his concerns: “The past experience from the Khmer Rouge haven't been properly solved or treated enough […] So, the way that can influence mostly or directly is parenting.”

Summarizing the findings on MECA domains, a picture of Cambodian clients' cultural identity emerges as a result from their socialization in hierarchical family structures with sociocentric values of a Buddhist‐influenced, collectivist culture. Nevertheless, these are shaped by other dimensions of intersectionality, such as the socioeconomic background, education, and geographic origin, which must be carefully considered to avoid stereotyping.

### 3.2 Context Conditions for Family Therapy

Several cultural barriers to access are apparent in the current findings. Religious, spiritual, and magical practices as traditional forms of counseling are present and determine notions of counseling in Cambodia. Sreyleak shares her observations: “Because even now we are living in the city but the most family still/when they have/something went wrong in their family the first thing they would like to meet with the monk or with the fortune teller or with someone that/about the spiritual.”

In the context of mental illness, counseling and therapy are highly stigmatized, reinforced by a culture of silence and the importance of protecting family honor and saving face, as Raksmey states:

Because there is a phrase of the/I think, the belief system of the tradition of Khmer people. They say, when we have something happen in our family just keep in the family not to bring it out to the outsider so that the other people will look down or laughing at our family. There is a pattern of the silence, to keep the problem silently.

The interviewees pointed out that basic care in the field of mental health has not been established due to a lack of financial resources and specialists. Several nongovernmental organizations are working with little coordination in this field. Decentralized outreach services and interventions that address community resources have proven to be viable in practice.

### 3.3 Practice Competencies Therapists Need to Bring to the Encounter With Families

In the following section, we present integrated results for the competencies identified by both interviewees and group discussion participants as important for working with families in the local context. While participants mentioned many basic counseling skills emphasized in Western models of therapy, there were cultural nuances in applying these skills to specific populations and family processes.

#### 3.3.1 Showing Respect for Elders in the Therapeutic Encounter

Empathy, active listening, building trust within a nonjudgmental, accepting therapeutic relationship were also named as fundamental for the Cambodian context. The interviewees referred to “basic counseling skills” as taught in the trainings they completed (cf. RUPP 2015). Thus, a fit of these generic competencies in Cambodia is evident but with a clear cultural accentuation of the respect that must be expressed in encounters with elderly clients. This significant attitude is expressed above all through the appreciation of family hierarchies in communication and respectful deference to elders, as Chenda explains in her statement:

But at the same time, I need to be creative or need to be flexible, how can I get that, how I show respect to the elder and then how can I get their attention to listen or to be able to work in the session with me. That is the art that I need to find a way to do that.

When addressing problematic behaviors, the clients' underlying good intentions for their actions should be elaborated first to counteract the culturally embedded mechanism of shame and loss of face through criticism. The extent to which the therapist can present herself as competent and trustworthy would also depend on how she is perceived as an expert and authority; in Cambodia, this would take place through the attribution of life experience and/or a high level of education.

#### 3.3.2 Addressing the Culture of Silence

Competencies need to be developed for meeting the strong cultural imperative not to discuss problems outside the family. The culture of silence and face‐saving protects the family's reputation from blame. This concern is met by a consistent solution‐focused assessment of strengths and resources, especially when problem descriptions are not the focus. Chenda explains:

And I think, the biggest and most important is to tackle the strengths and resources. I think, when we can help them to talk about strengths and resources more, it (is) really good to balance with the problem because if we focus only the presenting problem, there is only more problem, more problem. So, that is why/for me I feel like I love solution focused brief therapy, which I feel like is really applicable in Cambodian context with all surrounding challenges of stigmatization and the family don't have much time and all that stuff.

According to the training experts, the very reserved opening of the clients in the therapy process must therefore be respected—one rather expects a gradual opening with communication of information that gradually draws a more comprehensive picture of the (family) situation in the therapy or counseling process. The ability to create a safe discussion situation is important here (and not self‐evident in the—often improvised—settings). Therefore, one should not prematurely press for the participation of other family members.

#### 3.3.3 Meet Local Expectations for Healing

##### 3.3.3.1 Religion/Spirituality

The interviewees consider the competence to meet the religious beliefs of their clients in an informed and respectful way to be of particular importance, especially those religious/spiritual beliefs that are used to explain problems or mental disorders. Narith explains in his statement:

A lot of people until now still think that mental illness (is) not a problem that occurs by themself or by the chemical factor or by the neuron something like that. They only belief that this are the third power I mean that it's rather related to the traditional sector. I mean that from magic or from the Kruh, something like that.

In the interviews, the approach of traditional healers (Kruh Khmer) was succinctly summed up as the following triad by Sovannara; he stated: *“*Kruh Khmer refers to the person who can help to solve problems like you know, they listen to the family problem and then they can give advice and also they can give some herbs.”

##### 3.3.3.2 Expecting Direct Advice

The training experts stated that the expectation for advice must be met with appreciation, as this corresponds to the cultural imprint of the clients and with the precarious resources for long therapy processes. Different options could be offered; the clients could be guided by good questions and would then find solutions themselves. This requires well‐trained competencies in systemic questioning techniques according to the training experts. For relationship building purposes, the Cambodian therapists recommend adopting a both/and attitude regarding advice, as another option.

##### 3.3.3.3 Magical Beliefs

In the group discussion, the four training experts emphasized that it was important to gently meet the attribution of magical powers. In the Cambodian context, this is by no means meant metaphorically but ties in with the traditional expectations and theories about illness of those seeking advice, as the following example from the group discussion describes. The therapist had developed a solution construction with her client—on the path of exceptions from the problem in the past—within the framework of the solution‐oriented approach by using appropriate questioning‐based techniques. The client described this solution as extremely helpful for herself but attributed the source of the solution to the therapist's magical abilities according to her thinking habits. Chandra shares her encounter:

Yeah, she (client) believes that I am a magic person that can help her to think in the past, that she never thinks about that, so, she said: you are clever and like magic person, she said like: you click on my idea! She made a metaphor like I do like a magic stick on her brain (laughs).

The interviewed experts encountered different expectations from their clients, based on diverse world views. In the urban context, the prescription of medication is often expected since many clients assign the treatment of psychological problems to a biomedical model. Here, too, transitional phenomena can be seen in the transfer of magical ideas and familiar rituals—from taking medicinal herbs or substances with magical effects—to expectations of medicines and therapeutic offers in general. In addition to the abovementioned appreciation of the client's expectations, it is also important for the professionals interviewed to enable their clients to reflect on which are positive and which are harmful practices. In order to avert harm, cautious reference should be made to the alternative intervention strategies now offered. Somphoirs explains her approach:

And so, the therapists they must know this and not to disconnect people from culture or those ceremonies. […] But of course, we must be careful as a therapist whether it is an extreme attitude or extreme belief or not. We must find a way in the middle.

This includes asking about previous experiences with traditional treatment methods and about expectations in the current setting. The client's own convictions should not be questioned, but that reflection on previous experiences with traditional treatment should be encouraged. Sally puts it this way:

And whatever your fit or your belief system (is) I think, to support the person in being who they are and where they belong it/that's where it begins, we don't bring in our own, we support them where they are and their belief system is.

Then, new concepts can be offered about the possible causes of mental disorders or family problems. This strategy reflects the necessary competence of the Cambodian professionals to bridge between traditional and contemporary practices, not to overwhelm the clients with new practices and, to meet them in “their world”. In the interviews, it was emphasized that the beliefs of the clients should be respected because opposing ideas are not conducive to the healing process. Rather, it is beneficial to find commonalities with traditional approaches to healing, a caution against extreme practices, and finding a middle ground, an inclusive attitude.

#### 3.3.4 Integrate Into Local Structures and Activate Support Potentials

Networking with mental health services is very difficult because there are very limited structures of institutional support to which referrals can be made when needed. Sreyleak describes her challenges as a therapist: “In Cambodia, comparing to the west I think, it is quite different. Because when we are talking about the relevant stakeholders for example, supporting services, referral agencies, that in Cambodia we have limited places to connect to.” Forms of outreach work to often remote areas have been described. In order to gain access to clients in these communities, local authorities are first contacted. Panha describes his experiences:

First, we need to visit the village chief in the commune, […] we need to build a relationship with key people and explain them. […] When they know who we are they start to understand: […] we come to help people with mental health problems, so they are happy to help.

Both families and communities are involved, for example, in an ecological approach to the treatment of addictive disorders. The same applies when a family takes in foster children in the community. Likewise, outreach family therapy is conceivable in these assignments in remote villages. Sihak expresses his thoughts: “We still can work with a group of people in the community why not we adapt some kind of family therapy way to work. I think, we've been working in group already in the community.”

Families can be attracted to therapy if, for example, they can be shown the financial impact of family conflict and how to resolve it, as Chandra explains:

I think the skill to analyze, to link their problem to their economy (is needed) […] I think, if we have the skill to show them the conflict of the family can affect their economy—if they work on the conflict, economy also goes up—like that.

#### 3.3.5 Locally Necessary Systemic Competencies

##### 3.3.5.1 Trauma‐Informed Practices

The interviewed professionals named different aspects of systemic therapy, prominently the competence to work therapeutically with trauma in the context of the family, to address families with mentally ill family members, and to work on destructive behavioral and relational patterns such as substance abuse or domestic violence. In terms of networking, the four local expert colleagues emphasized in the group discussion that—unlike in Western countries—there is no network of diverse professional service providers in Cambodia for referring clients with corresponding needs. The main concern is to mobilize support within families or the community. In the analysis of the contextually determined different views of the participants, the international experts emphasized the family organization of the extended family present in Cambodia and described its support potential. It was also recommended by the experts interviewed that Buddhist or other religious concepts be incorporated into family therapy practice.

### 3.4 Reflective Competencies

The Cambodian training experts named competencies for self‐reflection, in the sense of how therapist's personal values, convictions, and professional self‐image must be taken into consideration. The interviewees emphasized that the imprint of one's own family system of origin should be reflected upon and the values of one's own culture should be accepted and often honored, but also critically questioned. This indicates respect for local knowledge along with a decolonizing attitude toward Western constructs, taught as universal.

Regarding the training of these reflexive competencies, the Cambodian training experts pointed out that self‐reflection is a new concept that must first be understood by the students. Therefore, the trainers should carefully guide this process. For cultural reasons, self‐reflection is misunderstood as an invitation to criticism or self‐criticism. Chenda states: “So, they are raised in a culture where disclosure is not good. We need to protect ourselves. So when they come to our training, and we ask them to disclose themselves—even we ensure confidentiality […] they still feel hesitant.” It is therefore crucial for the training experts to facilitate a new understanding of learning through reflection and guide the trainees to reflect on the values and convictions that clients bring and their cultural traditions and family contexts within which they move.

## 4 Discussion

There is no doubt that there are difficulties in providing psychosocial care for the Cambodian population as there is in other low‐ and middle‐income countries (Patterson et al. 2020), and therefore a comparison with Western care structures should be viewed critically (Charlés and Samarasinghe 2016). The questionable validity of Western, biomedical treatment standards should be taken into account here (White and Sashidharan 2014). The support systems available are primarily the family and the community in Cambodia, and skills are required to activate these (Patterson et al. 2020).

### 4.1 Bridging Different Belief and Value Systems

The results of this study show that Cambodian professionals generally encounter a variety of beliefs in their clients, very often magical–spiritual beliefs. Falicov (2009) identifies two basic points of reference in this context: (1) traditional indigenous syndromes based on spiritual beliefs about illness and healing and (2) beliefs in supernatural phenomena such as magic or magic cults. Both are inherent as cultural beliefs regarding causes of illness and cures for physical and mental suffering. In Cambodia, such indigenous syndromes include, among many others, baksbat (broken courage) as a culturally influenced trauma disorder or kut caraeun (thinking too much) as a pronounced stress symptomatology (van de Put and Eisenbruch 2002). In the statements of the Cambodian therapists, there is a tendency to distance themselves from indigenous (magical–animistic) approaches to their own therapeutic practice—for example, Chandra laughed at her client's magical beliefs. It is questionable to what extent the dominance of the Western canon of knowledge at the university (see RUPP 2015) has brought about this attitude and should therefore be seen as an effect of colonialism and epistemic violence (Brunner 2016).

For psychoeducation in Cambodia (as everywhere), it is important to inquire about existing concepts of illness and healing and to expand them gently in a way that clients and their families can follow while promoting self‐responsibility and coping. Furthermore, local culture and its canon of values also change over time as a result of social transformation processes; systems cannot not evolve (Borst and Ochs 2022). These transformation processes take place heterogeneously in Cambodian society for individuals and their families, depending on geographic location, education level, and age, as the results of this study point out. Due to these changes, different value systems come into conflict within families—such as between grandparents and parents in relation to parenting issues; between extended families and young couples in relation to living arrangements; or in relation to different gender norms within each generation or family (Falicov 2014b). Future family therapists must be equipped to understand and address these dynamics. All these considerations require extensive reflection, including self‐reflection.

### 4.2 Viewing Reflection as a Cultural Construct

Reflection as a human capacity articulates itself differently in culturally differently constructed modalities. Therefore, in transcultural situations, it is imperative to first ponder about reflection because it cannot be assumed that this process occurs in the same way in different cultures (Krause 2019). According to the training experts, self‐exploration and self‐assessment have been foreign to the Cambodian trainees so far; self‐reflection is not known as a learning format and has been misunderstood as an invitation to self‐criticism. There are also large macro or contextual issues. The formation of a collective professional identity as a counselor or a family therapist is nearly impossible in the absence of active professional associations and state recognition of the profession (Jordan et al. 2019). However, such professional organizations could be resources to reflect and integrate the personal and professional parts within “one's own group”. For the further development of the training in terms of reflection tasks, there is probably a lot of potential in designing peer group work or group supervision with rituals for self‐reflection.

### 4.3 Limitations

The current study has several limitations. The Royal University of Phnom Penh was asked to continue to support the training in an advisory capacity. This may have influenced the study not to consider the cultural adaptation of the training independent of its (current) location in the university context. This may also imply unspoken power issues. Furthermore, Cambodian families themselves were not included; the participants provided information from their perspective as experts. For future studies, families should be interviewed directly so that they have a platform to talk about themselves. An important question would be how families with mental health problems cope with them. In the survey on the current situation of Cambodian families and the problem areas in which family therapists work, race and power concerns as well as sociopolitical aspects were still insufficiently included and a topic for future research. A more in‐depth analysis of the contextually determined different perspectives of the participants would have been desirable, but this could not be implemented within the scope of this study. For example, what is it like for therapists to not only work with a traumatized population but to be traumatized themselves and having to provide services to populations that include the aggressors during the genocide? Due to the interviewer's limited language skills in Khmer, the interviews were conducted in English. This may have led to a loss in the quality of communication. However, the presence of a translator could possibly have influenced the openness of the interviewees.

## 5 Conclusions

In order to support culturally sensitive curricula for family therapy in Cambodia, local knowledge production was studied as central information in the development of basic cultural and contextual decolonial competencies needed here. For Cambodian family therapists, knowing and respecting the religious beliefs of the clients, being able to tie in with a culturally anchored understanding of disorders, as well as traditionally shaped ideas of therapy and healing emerged as essential. Considering the culture of silence and saving face and being able to deal respectfully with family hierarchies are necessary as well as paying attention to integration into local structures and activating support potentials that are accessible and sustainable: Those of the family and the community. For a responsive understanding of other people's culture, reflecting on one's own culture and thus recognizing differences and similarities can support the Cambodian experts in further adaptation work—local knowledge production—in relation to the Western‐influenced concepts of systemic family therapy. Future studies on self‐reflection of cultural influence are needed, for example, using the culture gram (Reibstein and Singh Reenee 2021) or MECA as a reflective practice. In the latter, the four MECA domains can provide the reflective framework here to allow trainees to explore their own cultural and contextual imprint as well as their preferences for certain theoretical models (Falicov 2014a, 2014b, 1995). The premise for this is clear: The “imported” or colonizing knowledge has emerged in completely different cultural contexts, so different value systems meet, and here it is necessary to establish fit—by local professionals as the experts of their own culture.

## 7 Conflicts of Interest

The authors declare no conflicts of interest.
